# Concerning Increase in Antimicrobial Resistance in Shiga Toxin-Producing *Escherichia coli* Isolated from Young Animals during 1980–2016

**DOI:** 10.1264/jsme2.ME17023

**Published:** 2017-09-27

**Authors:** Flore Chirila, Alexandra Tabaran, Nicodim Fit, George Nadas, Marian Mihaiu, Flaviu Tabaran, Cornel Cătoi, Oana Lucia Reget, Sorin Daniel Dan

**Affiliations:** 1 Microbiology Department, Faculty of Veterinary Medicine, University of Agricultural Sciences and Veterinary Medicine Manastur street no.3/5, Cluj-Napoca Romania; 2 Animal Breeding and Food Safety Department, Faculty of Veterinary Medicine, University of Agricultural Sciences and Veterinary Medicine Manastur street no.3/5, Cluj-Napoca Romania; 3 Pathologic Anatomy Department, Faculty of Veterinary Medicine, University of Agricultural Sciences and Veterinary Medicine Manastur street no.3/5, Cluj-Napoca Romania

**Keywords:** *E. coli*, Shiga toxin-producing *E. coli*, antibiotic resistance, health

## Abstract

This study was conducted in order to assess the antimicrobial resistance patterns of *E. coli* isolated from young animals affected between 1980 and 2016. The selected isolates for this study (*n*=175) carried *stx*_1_*/stx*_2_ genes and the most prevalent type of pathogenic *E. coli* found belonged to serogroup O101, antigen (K99)–F41 positive. All STEC-positive isolates were tested for susceptibility to 11 antimicrobials. Multidrug resistance (MDR) increased from 11% during the 1980s to 40% between 2000 and 2016. Resistance to tetracycline and streptomycin was the most frequent co-resistance phenotype (37%). Co-resistance to tetracycline and sulfonamide was found in 21% of *E. coli* isolates, while the MDR pattern to tetracycline, sulfonamide, and streptomycin was observed in 12% of the strains tested. Only 8% of isolates were co-resistant to tetracycline, ampicillin, streptomycin, and sulfonamide. The most common resistance genes found were those encoding for tetracycline, sulphonamides, and streptomycin, with 54% (*n*=95) of the tested isolates containing at least one of the genes encoding tetracycline resistance. A total of 87% of *E. coli* that tested positive for tetracycline (*tetA*, *tetB*, and *tetC*) and sulphonamide (*sul1*) resistance genes were isolated between 2000 and 2016. A large number of isolates (*n*=21) carried int1 and a nucleotide sequence analysis revealed that all class 1 integron gene cassettes carried *sul1*, *tet*, and *dfrA1* resistance genes. An increase was observed in the level of resistance to antimicrobials in Romania, highlighting the urgent need for a surveillance and prevention system for antimicrobial resistance in livestock in Eastern Europe.

Pathogenic *Escherichia coli* strains belong to the multiple bacteria responsible for the occurrence of enteric diseases (11). Based on their virulence scheme, *E. coli* isolates may be classified into six pathogroups: enterotoxigenic *E. coli* (ETEC), Shiga toxin-producing *E. coli* (STEC), enteropathogenic *E. coli* (EPEC), enteroinvasive *E. coli* (EIEC), enteroaggregative *E. coli* (EAggEC), and enteroadherent *E. coli* (EAdEC) (28, 44). ETEC infection is the most common type of colibacillosis in calves and piglets (47). Young animals are the most susceptible to ETEC infection during their first d of life, developing watery diarrhea if infected (22).

Commensal *E. coli* strains were susceptible to a large number of antimicrobial agents (32); however, due to the extensive use and uncontrolled treatment of farm animals (65, 66), antimicrobial-resistant strains have become a serious issue (66). The appropriate use of antimicrobial agents to treat neonatal diarrhea will be facilitated by a national database in which the most efficient antimicrobials are published according to their susceptibility. Although multiple studies have briefly described antimicrobial resistance and virulence factors in *E. coli* strains isolated from dairy calves in different regions worldwide (13, 23, 41), similar studies have yet to be performed in Romania. The most frequently prescribed antibiotic in human therapy in Romania is amoxicillin; however, similar to many other penicillins, the increasing resistance of bacteria has resulted in the need to discovery new therapies (20). Cephalosporin became available after the end of the communist era (its import had previously been forbidden) and its extensive use between 1990 and 2000 led to an increase in resistance, with therapies based on this active substance ultimately becoming inefficient. This has also occurred for erythromycin (20).

Aminopenicillins, chlortetracycline, and trimethoprim-sulphonamides are the typical antimicrobials used to treat diarrhea in animals; however, increasing antimicrobial resistance among *E. coli* isolates has led to the use of expanded-spectrum cephalosporin. Furthermore, a high prevalence of resistance to fluoroquinolones (FQs) has been observed in *Salmonella* cultured from meat and meat products in Romania; however, information on their current use in animals has not been provided (40).

The increased prevalence of antimicrobial resistance genes in isolates from food animals represents a growing concern (67). Many studies support fecal *E. coli* isolated from animals having the ability to infect humans directly and via food (45, 64). Bacteria may be intrinsically resistant to certain antibiotics, but may also acquire resistance to antibiotics via mutations in chromosomal genes and by horizontal gene transfer. Although the manner of acquisition of resistance may vary among bacterial species, resistance only develops via a few mechanisms: the acquisition of gene-encoding enzymes (*e.g.* β-lactamases) (60), increased activity of efflux pumps (59), acquisition of several genes that will lead to the production of bacterial cell walls lacking binding sites for antimicrobials, and the acquisition of mutations that will lead to decreased permeability (60). If these resistance genes are located on plasmids, they may be able to transfer rapidly among a number of bacterial species (38).

The horizontal transfer of resistance genes is a mechanism for the dissemination of multi-drug resistance (MDR) because resistance genes may be found in clusters and transferred together to the recipient (19). These genes may be transferred by different mechanisms of conjugation, transformation, or transduction. Under natural conditions, conjugation and transduction appear to be the most important transfer mechanisms in bacteria (56).

These mechanisms often involve integrons, which are mobile genetic elements that acquire antimicrobial resistance gene cassettes. Integrons are defined as genetic units that include determinants of the components of a site-specific recombination system capable of capturing mobile gene cassettes (3). The presence of class 1 integrons has been strongly correlated with MDR in *Enterobacteriaceae* (37).

This study aimed to investigate trends in resistance and antimicrobial resistance genes in 175 *E. coli* isolated from young animals (calves, foals, and piglets) with diarrhea symptoms in Romania during a 35-year period. The results of this study address the existing gap in resistance trends in *E. coli* isolates. This extended period of study will help to describe the evolution of resistance and emphasize the role of clinical medicine in the occurrence of resistant *E. coli*.

## Materials and Methods

### Samples and isolates

Samples were aseptically collected from calves, foals, and piglets by rectal swabbing, placed in sterile tubes, and transferred to the laboratory in a cool box within 3 h of collection. After arrival in the Microbiology Laboratory in the University of Agricultural Sciences and Veterinary Medicine Cluj, rectal swabs were transferred into separate tubes containing 2 mL nutrient broth and cultured at 37°C for one d following the steps stated in the ISO 16654:2001 protocol (26). Briefly, each sample was inoculated on MacConkey (Merck, Darmstadt, Germany) agar plates and incubated at 37°C overnight.

### Confirmation and selection of *E. coli* bacteria

All lactose-fermenting colonies were tested and considered positive after the application of an indole test. The indole test was made using Kovac’s reagent (0.5 mL) added to 2 mL of peptone water previously inoculated with 5 mL of bacterial growth. Samples were considered to be indole positive when a pink–red color developed within 15 min. Among the total number of *E. coli* strains identified (*n*=1346), only 175 were further subjected to susceptibility testing and the molecular characterization of resistance genes. The selection of these strains was based on serotype, the molecular identification of virulence genes, and clinical signs in animals (10).

### Serotyping and virulence gene identification

*E. coli* were serotyped based on the O (somatic lipopolysaccharide) antigen. The somatic antigen “O” was from lactose-positive strains (serogroup) and identified by serum agglutination in wells with monovalent anticolibacillar serum from O1–O157.

Multiplex PCR for detecting the genes encoding Shiga toxins (*stx_1_* and *stx_2_*), intimin (*eaeA*), heat-stable enterotoxin (*Sta*), heat-labile enterotoxin (LT), and the fimbriae of F41 and K99 was performed using the primers described in Table S1 (Supplementary file). The PCR assay was conducted in a final volume of 50 μL comprising the following: 1×PCR green Buffer, 1.5 mM MgCl_2_; 10 pmol of each primer, deoxynucleotides (dNTPs) each at 200 μM, 1.25 U of Taq DNA polymerase (Promega, Madison, WI, USA), and 5 μL of genomic DNA in a concentration of 50 ng μL^−1^. PCR was performed under the following conditions: 94°C for 3 min followed by 25 cycles of 94°C for 30 s, 54°C for 45 s, and 50°C for 1 min, and a final extension step of 70°C for 3 min. *E. coli* strains 152–2 (5) (*eae/stx*_1_*/stx*_2_) and ETEC23 (STa/LT-II/F41/K99) were used as positive controls, and *E. coli* DH5a was the negative control in all tests. Positive and negative controls were previously isolated in the Microbiology Laboratory in the University of Agricultural Sciences and Veterinary Medicine Cluj-Napoca.

### Susceptibility testing

The disk diffusion method of Bauer *et al.* (1966) (4) was used for susceptibility testing. Isolates were examined against several classes of antibiotics: Penicillins testing ampicillin (AMP, 10 μg), cephalosporins testing cefotaxime (CTX, 30 μg), ceftazidime (CAZ, 30 μg), macrolide testing chloramphenicol (CHL, 30 μg), quinolones testing ciprofloxacin (CIP, 5 μg) and nalidixic acid (NA, 30 μg), aminoglycoside testing gentamicin (GEN, 10 μg), streptomycin (S, 10 μg), sulphonamide testing sulfamethoxazole (SMX, 300 μg), trimethoprim/sulfamethoxazole (SXT, 1.25/23.75 μg), and tetracyclines (TET, 30 μg). The zones of inhibition were measured (including the diameter of the disk) using a ruler to the nearest millimeter and recorded. The *E. coli* ATCC 25922 strain was used as a quality control organism. Isolates were classified as “Resistant” or “Susceptible” based on the standard interpretation chart according to the Clinical Laboratory Standards Institute (12). Intermediate susceptible strains were considered susceptible in the interpretation of the results. We classified strains that showed resistance to three or more antimicrobials as MDR.

### PCR method for identification of resistance genes

All 175 strains were tested for the presence of S, GEN, sulfonamide, beta-lactams, erythromycin, TET, trimethoprim, and quinolone resistance determinants. In order to detect expressed and unexpressed genes, all isolates were screened, not taking into account their susceptibility profiles. The genes that were chosen for investigation were selected according to their prevalence among *E. coli*, which was previously suggested by other studies (17, 51).

DNA extraction followed a short protocol previously described by Mihaiu *et al.* (2014) (40). Briefly, 2–3 *E. coli* colonies growing on MacConkey media were removed with a sterile plastic loop and resuspended in 150 μL Chelex solution (Sigma Aldrich, St. Louis, MO, USA). The selection of specific *E. coli* colonies was based on lactose-fermenting properties and biochemical confirmation by the API 20E test (BioMerieux, Lyon, France). Sample tubes were subjected to high temperatures (94°C–15 min and 56°C–10 min). Assessments of the quantity and purity of DNA were made on a Nanodrop ND-1000 spectrophotometer analyzer (NanoDrop Technologies, Wilmington, DE, USA).

The identification of genes associated with resistance was performed by PCR and the sets of primers used are shown in Table S1 (Supplementary file). Eight classes of antimicrobials were investigated through the amplification of the following genes: aadA1 (S), *tet*(A), *tet*(B), *tet* (C), *tet*(G) (TET), *dfrA1* (trimethoprim), *qnrA* (quinolones), *aa*c (GEN), *sul1* (sulfonamides), *bla*_SHV_, *bla*_CMY_, *bla*_TEM,_ *bla*_CTX_ (beta-lactams), and *ere*(A) (erythromycin).

The PCR reaction mix (25 μL) comprised: 1×PCR green Buffer, 2.5 mM MgCl_2_; 5 pmol of each primer, dNTPs each at 200 μM, 2.5U of TaqDNA polymerase (Promega), and 100 ng of genomic DNA. Singleplex PCR was performed under the following conditions: 94°C for 3 min followed by 35 cycles of 94°C for 30 s, 58°C for 30 s, and 72°C for 1 min, and a final extension step of 73°C for 5 min. Following amplification, 10 μL from each PCR reaction containing the amplified product was loaded onto agarose gels (2%). The gels were stained with EvaGreen (JenaBioscience, Jena, Germany) and electrophoresed (90 W) for 40 min. Visualization was performed under UV light with a Gel Doc XR+Imager (Bio-Rad, Hercules, CA, USA). Strains of *E. coli* O157:K88ac:H19, CAPM 5933 and *E. coli* O159:H20, CAPM 6006 were used as positive controls.

### Integron class identification and characterization

The presence of class 1 and class 2 integrons was analyzed by the PCR amplification of integrase genes (*int1* and *int2*), variable regions containing gene cassettes (*c1gc* and *c2gc*), and the 3′ conserved region of the class 1 integron (*qacEΔ1* and *sul1*) (29).

Amplification products were extracted from gels with the FavorPrep^TM^ Gel/PCR Purification Kit (Favorgen, Ping-Tung, Taiwan). The amplified products were sequenced in an authorized laboratory (Macrogen, Seoul, Korea) with the class 1 and class 2 integron forward and reverse primers (Table 1). In order to identify gene cassettes in integrons, each gene cassette region in classes 1 and 2 was amplified and further sequenced. Sequence alignments were confirmed using the GenBank database and BLASTX search engine (2).

### Statistical analysis

The Mann-Kendall test was performed to identify increasing or decreasing trends over the period studied. The magnitude of change over the 35-year period was estimated using a slope parameter (Q), and the Sen non-parametric method, according to a previously described protocol (52). The 35-year period was analyzed according to three time intervals (1980–1989; 1990–1999; 2000–2016) in order to obtain sufficient data for statistical interpretations of possible changes in resistance. Between 1980 and 1989, 35 isolates were investigated, while 70 samples per period were analyzed in the other two time intervals. Significance was assessed at *P*<0.05.

## Results

### Serotype and virulence gene characterization

The results of serological identification are shown in Table 2. The isolates selected for this study (*n*=175) carried *stx*_1_/*stx*_2_ genes. This number represents a significant percent (13%) from the total amount of samples collected between 1980 and 2016 (*n*=1346), which tested positive in the presence of *E. coli*. The most prevalent type of pathogenic *E. coli* found belonged to serogroup O101, antigen (K99)–F41 positive. A total of 20 (11%) strains were positive for *eae* and were more frequently detected between 2000 and 2016 (11/175, 6%) than between 1980 and 1989 (4/175, 2%; *P*<0.001). The *eae*-positive strains were positive for *stx*_1_ (13 isolates) and *stx*_2_ (7 isolates) in some cases. The genes for LT-I and LT-II were detected at a markedly higher percentage between 2000 and 2016 (5%; 10 isolates) than between 1980 and 1989 (1%; 3 isolates; *P*<0.001). All of the strains that were positive for LT genes were positive for F5. Regarding *STa* genes, 17 isolates (9%) were found to be positive by PCR, with 7 being positive for F5 and 10 for F41 (Table 2). Of the 175 strains of *E. coli* analyzed, 64 were not typeable, 28 were rugous, and 83 belonged to 8 different serogroups (Table 2). One hundred and twenty *stx*-positive isolates were negative for the *eae*, *LT*, and *STa* genes.

### Antimicrobial susceptibility of isolates

The antimicrobial susceptibility profile showed that 108 of the isolates tested (61.7%) were resistant to one or more antimicrobials. The most common resistance phenotypes were to older antimicrobials such as TET (64%), sulfonamide (44%), S (24%), and AMP (22%). *E. coli* remained the most susceptible to cephalosporins and quinolones with 5% and 3%, respectively, showing a resistance phenotype (Table 1).

The proportion of *E. coli* isolates susceptible to all antibiotics showed a decrease from 60% between 1980 and 1989 (*n*=21) to 38% between 2000 and 2016 (*n*=26). The lowest percentage of susceptible strains (11%) was found between 1990 and 1999 (*n*=20). Conversely, MDR increased from 11.4% between 1980 and 1989 (*n*=4) to 40% between 2000 and 2016 (*n*=28). Significant changes in resistance patterns (*P*<0.001) were noted when comparing the isolates collected in 1980s with those from the 1990s and 2000s. This significant increase was observed for TET, sulfonamide, and S. Regarding the other classes of antimicrobials studied, no significant changes were noted; however, a slight increase was found.

Seventy-four (43%) *E. coli* isolates showed MDR phenotypes, with 19 (10.8%) exhibiting resistance to 4 or more antimicrobial classes. A larger proportion of these MDR isolates was recovered between 2000 and 2016 (*n*=34) than between 1980 and 1989, during which only 6 isolates were MDR. Between 1990 and 1999, we isolated 34 strains that showed MDR phenotypes. No significant difference (Mann-Kendall test) (*P*>0.05) was noted when the number of MDR phenotypes isolated between 1990 and 1999 was compared with that isolated between 2000 and 2016. Two strains showed resistance to all the antimicrobials tested and both of them were recovered in 2004.

The most common co-resistance phenotypes identified were TET/S (37%) (Table 1), followed by TET/sulfonamide (21%) and TET/S/sulfonamide (12%).

Resistance to cephalosporin and sulphamethoxazole/trimethoprim was rare and only found in isolates that showed MDR. None of the isolates collected between 1980 and 1990 showed resistance to cephalosporin and only one isolate was resistant to sulphamethoxazole/trimethoprim. Three isolates collected between 2000 and 2016 showed concurrent resistance to cephalosporin and sulphamethoxazole/trimethoprim.

In the present study, GEN resistance was not observed in isolates collected in the 1980s. In the 1990s, only one isolate was resistant to GEN, and this number increased between 2007 and 2016 to 6 isolates.

### Prevalence of resistance genes

The most common resistance genes found were those encoding for TET, sulphonamide, and S resistance. A total of 54% (*n*=95) of the tested isolates contained at least one of the genes encoding TET resistance, with the *tetA* gene being the most prevalent (48%; *n*=84). Three out of the 95 isolates that carried TET genes were not phenotypically resistant to TET. The genes *tetB* and *tetC* were also identified at a high proportion (38% and 24%, respectively). Genes responsible for resistance to sulfonamides (*sul1*) and trimethoprim (*dfrA1*) were detected in 22% (*n*=40) and 18% (*n*=33), respectively, of the isolates. We found that 87% of *E. coli* that tested positive for TET (*tetA*, *tetB*, and *tetC*) and sulphonamide (*sul1*) resistance genes were isolated between 2000 and 2016. This resistance to sulphonamide was observed in six isolates (11%) between 1980 and 1990, while this number markedly increased (64%) between 2010 and 2016. Furthermore, 29% of *E. coli* that carried the beta-lactamase (*bla*_TEM_) gene was isolated in the 2000s. None of the *E. coli* isolated within the 1980s tested positive for beta-lactam or quinolone genes (Table 3).

According to our statistical analysis, the prevalence of TET, trimethoprim, sulfonamide, and beta-lactamase genes was significantly higher (*P*<0.001) between 2000 and 2016 than in the 1980s.

### Integron characteristics

Among the *E. coli* isolates investigated (*n*=175), 23 (13%) showed integrase-positive results. Among these, 21 (91%) carried *int1*, 4 (17%) *int2*, and 2 (8%) *int1* and *int2*. Collectively, they accounted for 48% of the 47 MDR isolates characterized in the present study. Our results also revealed a higher prevalence of integron-positive *E. coli* isolated between 2000 and 2016 (*n*=15; 71%).

The nucleotide sequence analysis revealed that all class 1 integron gene cassettes carried *sul1*, *tet*, and *dfrA1* resistance genes. Four gene cassettes that encoded resistance to trimethoprim (*dfrA1*), S (*aadA1*), and betalactams (*bla*_SHV_ and *bla*_TEM_) were detected in five class 1 integrons. Two different types of class 2 integrons, *dfrA1*-*aadA1* and (*bla*_TEM_-*aadA1*), were found in int2-positive isolates.

## Discussion

The major goal of the present study was to document antimicrobial resistance patterns among *E. coli* isolates that caused neonatal diarrhea in young calves born between 1980 and 2016 in Romania. This study is important because almost 25% of calves are affected by diarrhea before weaning (58), making it the number one cause of morbidity and mortality (61). A previous study on European production animal systems, which did not include Romania, reported very high levels of antimicrobial resistance in veal farms (46), indicating that these units are a key target in risk reduction strategies (16).

We confirmed that veal farms represent an important reservoir of resistant bacteria because the pathogenic *E. coli* isolated exhibited increased resistance to 6 antimicrobial agents, including AMP, sulfonamide, TET, SXT, and S, which is a cause for concern due to the implied hazards. Meat quality is constantly monitored in Romania to ensure there are no residues in meat; however, there is a risk that drug-resistant strains are passed on through direct contact between humans and animals (notably farmers). There is a further indirect threat to human health as a result of animal excretion, resulting in the release of resistant bacteria into the environment, thereby providing opportunities for exposure and the creation of selective pressure for the development of antimicrobial resistance (62).

The present study revealed a high prevalence of resistance to TET, sulfonamide, and AMP. This pattern of resistance has increased over time and may have occurred due to the irresponsible use of these antimicrobials in animal treatments for different infections (21). A previous study reported the yearly use of 300,000 kg of antimicrobials in some countries as veterinary prescriptions for animals (63).

In contrast to other studies, our results on the resistance of *E. coli* isolates from neonatal diarrhea-affected calves showed a markedly lower trend (49, 69). This pattern difference may reflect the various uses of antimicrobial agents in different regions and countries. Due to the high resistance rate of *E. coli* isolated between 2010 and 2016 to AMP, S, and TET, the phenotypes of resistance have increased from those between 1980 and 1989. The increase in antimicrobial resistance to these agents over time may be explained by some (TET, S, penicillin, and sulfonamides) being added to calf milk replacer in order to combat the stresses faced early in life (7). This practice was previously performed in an attempt to delay morbidity and decrease mortality (6) as well as promote increased growth and improve feed efficiency (8).

Previous studies reported a link between the level of antimicrobial usage in growth units and acquired resistance in *E. coli* in calves (14, 27, 68). Therefore, antimicrobial therapy in animals may be the cause of the high level of resistant bacteria diffused in the farm environment (6). The age-related occurrence of resistance has also been suggested. *E. coli* serotypes isolated from older calves were found to have different resistance patterns from those from younger calves (25). This finding indicates that the shift observed in resistance patterns is due to the acquisition of new resistant strains rather than to the reestablishment of the same strains (30). Our study focused on young aged animals (2–4 months old) bred in the same environment and growth systems. We considered contamination to be influenced by diet and resistance to be the highest in the milk-feeding period (17).

Resistance to sulfonamide was one of the most common resistance profiles identified, and also showed the greatest increase over time. Sulfonamides were introduced in the 1930s and have since been used as therapy. In veterinary medicine, sulfonamide is one of the most commonly used antimicrobials (39) and a high prevalence of clinical resistance has been reported in bacteria isolated from humans and food products (19, 21, 31). This phenomenon is often associated with the presence of *sul* genes (31). Sulfonamide resistance genes are also associated with mobile genetic elements (integrons), which play a major role in the dissemination of multiple antimicrobial resistance genes (5).

A corresponding resistance gene was detected in most phenotypically resistant strains. Under conditions in which the gene was not identified, a possible explanation was provided by the limited number of genes tested in this study. This study also showed that resistance genes were identified in phenotypically susceptible strains. This result may be explained by a possible lack of expression in these bacteria. To the best of our knowledge, this study is the first to describe the antimicrobial resistance patterns and genes involved in *E. coli* causing neonatal diarrhea in young calves in Romania. Among 175 *E. coli* isolates, 112 were resistant to at least one antimicrobial tested and 37 were classified as MDR.

The identification of a high proportion of *tetA* and/or *tetB* genes in TET-resistant isolates indicates that the main mechanism of TET resistance in calf *E. coli* isolates is through active efflux (70). A predominance of the *tetB* gene has been observed among TET-doxycycline-resistant *E. coli* isolates of diarrheic calves, which is consistent with previous findings obtained in different countries (15, 53). Resistance genes were carried by phenotypically susceptible strains of bacteria, which may have led to their expression not yet occurring. Other studies have also shown the lack of expression of resistance genes in some bacteria (33, 50, 57).

Integron genes in *Enterobacteriaceae* are widespread and contribute to MDR (36, 37). Class 1 and class 2 integron genes were found within our *E. coli* isolates. Their prevalence was lower than that previously reported in *E. coli* isolated from poultry and pigs (34). In the present study, 13% of *stx*-positive strains were also positive for integrons. Previous studies revealed a higher prevalence of integron-positive STEC strains isolated in the USA and originating from human patients (*n*=81) and sick animals (*n*=193; poultry, cattle, and swine) (54). Zhao *et al.* (2001) (71) revealed an integron class 1 prevalence of 18% in 50 analyzed STEC strains originating from humans, animals, and food. Our results revealed the lack of marked variations in the antimicrobial-resistance gene cassettes present in the two different integron types. They all contained genes coding for resistance to S/spectinomycin (*aadA1*) and/or trimethoprim (*dfrA1*). A study performed in Brazil on thirty-two STEC strains showed that the integrase gene associated with class 1 integrons in 22% of isolates, all of which had a uniform size and contained a single gene cassette, *aad*A1 (S resistance) (9). Povilonis *et al.* (2010) (48) described similar findings to the present results, showing that the most prevalent integron types contained the arrays *dfr*A1-*aad*A1 (36%), *dfr*A17-*aad*A5 (23%), and *dfr*A1-*sat*1-*aad*A1 (78%), while Skurnik *et al.* (2005) (55) revealed that 85% of class 1 integrons carried the *dfr* and/or *aadA* genes. Our results showed that the most common gene cassette array was *dfrA1*, which was present in 21 of the integron 1-positive strains. Most of the integron-positive strains (87%) were resistant to at least three different antimicrobials and their prevalence was higher between 2000 and 2016 (*P*<0.001) than between 1980 and 1989. In integron-negative strains, 23% were resistant to at least three antimicrobials and were isolated at a higher frequency between 1989 and 1999 (*P*<0.001) than between 1980 and 1989. Our results were consistent with previous findings reported by Nagachinta & Chen (2009) (43), who showed that all integron-positive strains examined were resistant to at least three different antimicrobials. Integron-positive strains were significantly more resistant to TET (82%), sulfonamides (65%,) and trimethoprim (46%) than integron-negative strains. The strains isolated between 2000 and 2016 showed a higher prevalence of integron classes, which is a clear warning signal for the rapid spread of antimicrobial resistance.

Another factor that is a cause for concern is that all integron-positive strains showed virulence factors that are relevant because neonatal diarrhea produced by *E. coli* strains is an important cause of economic losses on farms. Most of the isolates showing the presence of these virulence factors were isolated between 2000 and 2016, which is concerning and draws attention to not only *E. coli* becoming more resistant to antimicrobials, but also more aggressive. *E. coli* isolated from animals may also present genes that are related to virulent strains in humans (35). In this study, we identified 5 strains with the gene for LT-II, which was unexpected because it is not common in calves (42).

*E. coli* producing Shiga toxins and carrying the *eae* gene are commonly isolated from the feces of calves and cattle (1); however, we only found twenty isolates that carried the *eae* gene. The presence of the *stx*_1_ and *stx*_2_ genes in 13% of the analyzed *E. coli* isolates is consistent with that reported by Güler *et al.* (2008) (24) in Turkey, revealing that 13% and 5% of *E. coli* isolated from diarrheic calves presented *stx*_1_ and *stx*_2_, respectively.

The STEC strains of *E. coli* showed markedly high resistance patterns, particularly between 2000 and 2016, which is very important because the Stx toxin may be involved in economic losses and also has a large impact on human health because these animals may be carriers to humans. Cattle are considered to be a major reservoir of STEC worldwide (1); however, field literature indicates that regions with a high prevalence of STEC in dairy cattle typically do not have a high incidence of cases of human infections (42).

## Conclusion

STEC-positive *E. coli* isolated from calves affected by neonatal diarrhea showed a high level of resistance to currently used antimicrobials. The results of the present study, which were obtained over a period of 35 years, suggest concerning increases in resistant STEC strains in young calves affected by neonatal diarrhea. The high rate of STEC isolation, diversity of ETEC serogroups, and presence of virulence factors described here identify dairy calves as important reservoirs of STEC and ETEC in our setting. This analysis provides information regarding the development of resistance over time, which lays the groundwork for understanding the evolution of MDR at the genetic level in Romania. Most MDR *E. coli* serogroups are associated with human diseases, which suggests a potential source of public health and environmental spread in the European community.

## Supplementary Material



## Figures and Tables

**Table 1:** *E. coli* resistance patterns

Co-resistance pattern	No. (%) of isolates
TET, STREP	37 (21)
AMP, TET, STREP	21 (12)
CF, QUIN, OXT	2 (1)
AMP, TET, STREP, SXT	9 (5)
TET, AMP, QUIN, OXT, GEN	3 (1)
AMP, STREP, OXT, CF, QUIN, GEN	3 (1)
AMP, CF, STREP, OXT, SXT, GEN	1 (0.5)
AMP, TET, CF, STREP, OXT, NA, GEN	1 (0.5)
AMP, CF, TET, STREP, OXT, SXT, GEN	2 (1)
Others^a^	34 (19)
No resistance	63 (36)
Susceptible	

a Isolates with two or less antimicrobial agents.

**Table 2:** Hemolytic and fimbrial adhesin-producing*E. coli* strains isolated from young animals with diarrhea and positive for *Stx*

Toxins	No. (%) of *E. coli* strains			Serogroups (no. isolates)

	Period			

	1980–1989	1990–1999	2000–2016	
LT-I and *Stx-1*	3 (1.7)	12 (6.8)	5 (2.8)	O1(1), O17(1), O23(3), O78(1), O139(11), O146(1), O153(2)
LT-II and *Stx-1*	5 (2.8)	16 (9.1)	21 (12)	O23(4), O26(3), O55(2), O103(4), O117(5), O123(1), O124(6), O144(3), O146(7), ONT(1), Rugous(6)
LT-I and *Stx-2*	0	1 (0.55)	2 (1.1)	Rugous(3)
LT-II and *Stx-2*	0	0	6 (3)	O1(1), O10(1), O103(2), ONT(1), Rugous(1)
Sta and *Stx-1*	4 (2.2)	2 (1.1)	3 (1.7)	O103(2), O117(2), O123(1), O124(1), O144(1), Rugous(2)
Sta and *Stx-2*	2 (1.1)	1 (0.5)	5 (2.8)	O144(3), Rugous(5)
Eae and *Stx-1*	4 (2.2)	3 (1.7)	6 (3.4)	O23(2), O26(3), O55(2), O103(2), O117(1), O123(3)
Eae and *Stx-2*	0	2 (1.1)	5 (2.8)	O146(1), ONT(1), Rugous(5)
Fimbriae				
F5 positive	14 (8)	54 (30.8)	42 (24)	O23(6), O26(6), O55(4), O103(4), O117(15), O123(11), O124(6), O144(3), O146(7), ONT(1), Rugous(16), N* (41)
F41 positive	2 (1.1)	23 (13.1)	25 (14.2)	O103(2), O117(5), O123(3), O124(1), O144(5), Rugous(12), N* (22)

* N—not typed.

**Table 3:** Phenotypes and resistance genes detected

Phenotype of resistance	Resistance genes detected
TET, STREP	TetA, aadA1
AMP, TET, STREP	blaTEM, tet A, tetB, tetC, aadA1
CF, QUIN, OXT	dfrA1, qnrA, tet A, tetB, bla_SHV_
AMP, TET, STREP, SXT	blaTEM, tet A, tetB, tetC, aadA1, dfrA1
TET, AMP, QUIN, OXT, GEN	tet A, tetB, tetC, blaTEM, sul1
AMP, STREP, OXT, CF, QUIN, GEN	sul1, tet A, tetC, blaTEM, sul1
AMP, CF, STREP, OXT, SXT, GEN	blaTEM, tet A, tetB, tetC, aadA1, dfrA1
AMP, TET, CF, STREP, OXT, NA, GEN	blaTEM, tet A, aadA1, dfrA1, ereA
AMP, CF, TET, STREP, OXT, SXT, GEN	blaTEM, tet A, tetB, aadA1, dfrA1
