# Supplementary material for: Concerning Increase in Antimicrobial Resistance in Shiga Toxin-Producing Escherichia coli Isolated from Young Animals during 1980–2016
Source: Microbes Environ. 2017 Sep 27;32(3):252–9. doi: 10.1264/jsme2.ME17023 (PMC5606695; doi:10.1264/jsme2.ME17023)
Supplement: Supplementary file 1 [file 32_252_s1.pdf]

Table S1 (Supplementary file)

PCR primers used for the PCR

Primer	Sequence (5-3)	Size of amplified product	Annealing temperature	Reference
eae	GACCCGGCAACAAGCATAAGC<br>CCACCTGCAGACAACAAGAGG	384	55	2
LT-I	TATCCTCTCTATATGCACAG<br>CTGTAGTGGAAGCTGTTATA	480	50	2
LT-II	AGATATAATGATCCATATGTATC<br>TAACCCTCGAAATAAATCTC	300	50	5
STa	TCCGTGAAACAACATGACGG<br>ATAACATCCAGCACAGGCAG	244	58	8
Stx-1	AGGTGCAGCTCTCTTTCAATA<br>TGCAAACAAATTATCCCCTGAG	364	57	2
Stx-2	GGGCAGTTATTTTGCTGTGGA<br>GTATCTGCCTGAAGCGTAA	386	57	2
F5 (K99)	TGGGACTACCAATGCTTCTG<br>TATCCACCATTAGACGGAGC	450	58	3
F41	GAGGGACTTTCATCTTTTAG<br>AGTCCATTCCATTTATCGGC	431	56	3
aadA1	TATCCAGCTAAGCGCGAAC<br>ATTTGCCGACTACCTTGGT	447	58	10
aac(3) - IV	CTTCAGGATGGCAAGTTGGT<br>ATGATCTAACCCTCGGTCTC	286	55	10
sul1	TTCGGCATTCTGAATCTCAC<br>ATGATCTAACCCTCGGTCTC	822	47	10
bla_{SHV}	TCGCCTCTCTATTATCTCCC<br>CGCAGATAAATCACCACAAT	768	52	10
bla_{CMY}	TGGCCAGAACTGACAGGCAAA<br>TTTCTCCTGAACGTGGCTGGC	462	47	10
bla_{TEM}	ATTCTTGAAGACGAAAGGGC<br>ACGCTCAGTGGAACGAAAAC	1150	60	6
ereA	GCCGGTGCTCATGAACTTGAG<br>CGACTCTATTCGATCAGAGGC	419	52	10
tetA	GGTTCACTCGAACGACGTCA<br>CTGTCCGACAAGTTGCATGA	577	57	6
tetB	CCTCAGCTTCTCAACGCGTG<br>GCACCTTGCTGATGACTCTT	634	56	6
tetC	TCTAACAATGCGCTCATCGT<br>GGTTGAAGGCTCTCAAGGGC	570	62	4
tetG	ATTACACTGCTGGACGCGAT<br>CTGATCAGCAGACAGATTGC	1104	57	4
dfrA1	GGAGTGCCAAAGGTGAACAGC<br>GAGGCGAAGTCTTGGGTAAAAAC	367	45	9
qnrA	GGGTATGGATATTATTGATAAAG<br>CTAATCCGGCAGCACTATTTA	670	50	9
Int1	GGGTCAAGGATCTGGATTTCG<br>ACATGGGTGTAAATCATCGTC	484	58	1
Int2	CACGGATATGCGACAAAAAGGT<br>GTAGCAAACGAGTGACGAAATG	789	54	1

4

Table S2 (Supplementary file)
STEC Isolate characterization

Isolate	Year of isolation	Virulence gene	Antimicrobial resistance	Host animal	Serogoup
**STEC1**	1982	*Stx-1 (LT-I)*	TET, STREP	calf	O23
**STEC2**	1983	*Stx1 (LT-1)*	AMP, TET, STREP	calf	O78
**STEC3**	1990	*Stx1* (LT-II)	AMP, TET, STREP	calf	O23
**STEC4**	1990	*Stx1* (LT-II)	AMP, TET, STREP	calf	O23
**STEC5**	1991	*Stx1 (LT-I)*	AMP, TET, STREP	calf	O23
**STEC6**	1984	*Stx1 (Sta)*	TET, STREP	calf	Rugous
**STEC7**	1994	*Stx1* (*Eae*)	TET, AMP, QUIN, OXT, GEN	calf	O117
**STEC8**	1995	*Stx 1*	Susceptible	calf	O101
**STEC9**	1996	*Stx 2*	Susceptible	calf	O117
**STEC10**	1993	*Stx1; Stx2*	TET, STREP	calf	O144
**STEC11**	1996	*Stx1*	TET, STREP	calf	O123
**STEC12**	1998	*Stx1* (LT-II)	AMP	calf	Rugous
**STEC13**	1999	*Stx2* (*Eae*)	AMP	calf	ONT
**STEC14**	1983	*Stx1 (Sta)*	AMP	calf	Rugous
**STEC15**	1983	*Stx1*	AMP	calf	O123
**STEC16**	1984	*Stx1* (LT-II)	AMP	calf	O23
**STEC17**	1983	*Stx1* (LT-II)	TET, AMP, QUIN, OXT, GEN	calf	Rugous
**STEC18**	1986	*Stx1*	Susceptible	calf	O146
**STEC19**	1988	*Stx1* (*Eae*)	AMP, TET, STREP	calf	O123
**STEC20**	1982	*Stx1* (*Eae*)	AMP, TET, STREP	calf	O123
**STEC21**	1994	*Stx1*	AMP, TET, STREP	calf	O153
**STEC22**	1995	*Stx1* (*Eae*)	Susceptible	calf	O123
**STEC23**	1996	*Stx1*	TET, AMP, QUIN, OXT, GEN	calf	O146
**STEC24**	1993	*Stx1*	Susceptible	calf	O124
**STEC25**	1996	*Stx1 (LT-1)*	Susceptible	calf	O139
**STEC26**	1998	*Stx1* (LT-II)	Susceptible	calf	Rugous
**STEC27**	1999	*Stx1*	Susceptible	calf	O101
**STEC28**	1994	*Stx1*	Susceptible	calf	O146
**STEC29**	1995	*Stx1 (LT-1)*	Susceptible	calf	O139
**STEC30**	1996	*Stx1* (LT-II)	Susceptible	calf	O139
**STEC31**	1993	*Stx1* (LT-II)	TET[a], STREP[b]	calf	O23
**STEC32**	1996	*Stx1 (LT-1)*	TET	calf	O139
**STEC33**	2006	*Stx1*	TET[a], STREP[b]	calf	O146
**STEC34**	2002	*Stx1* (LT-II)	TET[a], STREP[b]	calf	O117
**STEC35**	2002	*Stx1* (LT-II)	AMP, TET, STREP	calf	O117
**STEC36**	2003	*Stx1* (*Eae*)	TET, AMP, QUIN, OXT, GEN	calf	O103
**STEC37**	2008	*Stx1*	TET, STREP	calf	O139
**STEC38**	2006	*Stx1 (LT-1)*	TET, STREP	calf	O139
**STEC39**	2002	*Stx1* (LT-II)	AMP, TET, STREP	calf	O117
**STEC40**	2002	*Stx1* (*Eae*)	TET, STREP	calf	O103
**STEC41**	2005	*Stx1 (LT-1)*	TET, STREP	calf	O139
**STEC42**	2008	*Stx1*	TET	calf	O101
**STEC43**	2001	*Stx1 (LT-1)*	TET	calf	O139
**STEC44**	2001	*Stx1*	TET, STREP	calf	O117
**STEC45**	2001	*Stx1*	TET, STREP	calf	O103

**STEC46**	2008	*Stx1(LT-1)*	TET, STREP	calf	O124
**STEC47**	2007	*Stx1*	AMP, TET, STREP	calf	O146
**STEC48**	2002	*Stx1(LT-1)*	TET, STREP	calf	O139
**STEC49**	2009	*Stx1 (LT-1)*	TET, STREP	calf	O139
**STEC50**	2001	*Stx2 (Sta)*	AMP, TET, STREP	calf	Rugous
**STEC51**	2008	*Stx2 (Sta)*	TET, STREP	calf	Rugous
**STEC52**	2001	*Stx2 (Sta)*	TET, AMP, QUIN, OXT, GEN	calf	Rugous
**STEC53**	1983	*Stx2 (Sta)*	Susceptible	piglet	Rugous
**STEC54**	1986	*Stx1(LT-1)*	TET	piglet	O139
**STEC55**	1988	*Stx1*	Susceptible	piglet	ONT
**STEC56**	1982	*Stx1(LT-1)*	Susceptible	piglet	O117
**STEC57**	1983	*Stx1*	Susceptible	piglet	O139
**STEC58**	1986	*Stx1*	Susceptible	piglet	O139
**STEC59**	1988	*Stx1* (LT-II)	TET, STREP	piglet	O117
**STEC60**	1982	*Stx1* (LT-II)	TET, STREP	piglet	O117
**STEC61**	1994	*Stx1*	TET, STREP	piglet	O101
**STEC62**	1995	*Stx1* (*Eae*)	TET, STREP	piglet	O55
**STEC63**	1996	*Stx1*	AMP, TET, STREP	piglet	O146
**STEC64**	1993	*Stx1*	AMP, TET, STREP	piglet	O153
**STEC65**	1996	*Stx1* (LT-II)	AMP, TET, STREP	piglet	O117
**STEC66**	1998	*Stx1*	TET, AMP, QUIN, OXT, GEN	piglet	O1
**STEC67**	1999	*Stx1* (LT-II)	TET, STREP	piglet	O10
**STEC68**	1994	*Stx1(LT-1)*	TET, STREP	piglet	O123
**STEC69**	1995	*Stx1* (*Eae*)	TET, STREP	foal	O55
**STEC70**	1996	*Stx1*	TET, STREP	foal	O146
**STEC71**	1993	*Stx1(LT-1)*	AMP, TET, STREP, SXT	foal	O139
**STEC72**	1996	*Stx1*	AMP, TET, STREP	calf	O101
**STEC73**	2003	*Stx1*	AMP, TET, STREP	calf	O101
**STEC74**	2008	*Stx1 (LT-1)*	AMP, TET, STREP, SXT	calf	O101
**STEC75**	2006	*Stx1* (LT-II)	TET, STREP	calf	O101
**STEC76**	2002	*Stx1*	TET, STREP	calf	O101
**STEC77**	2002	*Stx1 (LT-1)*	TET, STREP	calf	O153
**STEC78**	2005	*Stx1 (LT-1)*	TET, STREP	calf	O153
**STEC79**	1991	*Stx1 (LT-1)*	Susceptible	calf	O146
**STEC80**	1984	*Stx1* (*Eae*)	Susceptible	calf	O26
**STEC81**	1994	*Stx1*	Susceptible	calf	O117
**STEC82**	1995	*Stx1*	TET, AMP, QUIN, OXT, GEN	calf	O139
**STEC83**	1996	*Stx1* (LT-II)	Susceptible	calf	O139
**STEC84**	2003	*Stx1* (*Eae*)	TET	calf	O26
**STEC85**	2008	*Stx1*	TET, AMP, QUIN, OXT, GEN	calf	O101
**STEC86**	2006	*Stx1*	TET,GEN	calf	O101
**STEC87**	2002	*Stx1*	TET,AMP	calf	0101
**STEC88**	1983	*Stx1*	TET	calf	O103
**STEC89**	1984	*Stx1*	Susceptible	calf	O124
**STEC90**	1984	*Stx1* (LT-II)	Susceptible	calf	O146
**STEC91**	1982	*Stx1*	Susceptible	calf	O146
**STEC92**	1994	*Stx1*	Susceptible	calf	Rugous
**STEC93**	1995	*Stx1* (LT-II)	TET, STREP	calf	Rugous
**STEC94**	1996	*Stx1* (LT-II)	TET, STREP	calf	Rugous

**STEC95**	1993	*Stx2 (Sta)*	TET, STREP	calf	Rugous
**STEC96**	1996	*Stx1*	TET, STREP	calf	Rugous
**STEC97**	1998	*Stx1*	Susceptible	calf	Rugous
**STEC98**	1999	*Stx2* (*Eae*)	AMP, TET, STREP, SXT	calf	ONT
**STEC99**	1994	*Stx1*	Susceptible	calf	O117
**STEC100**	1995	*Stx1*	Susceptible	calf	O139
**STEC101**	1996	*Stx1*	Susceptible	calf	O139
**STEC102**	1993	*Stx1 (Sta)*	TET, STREP	calf	O103
**STEC103**	1996	*Stx2* (*Eae*)	TET, STREP	calf	O101
**STEC104**	2003	*Stx1*	TET, SXT	calf	O101
**STEC105**	2008	*Stx1* (LT-II)	TET, SXT	calf	O101
**STEC106**	2006	*Stx1*	TET, SXT	calf	O146
**STEC107**	2002	*Stx1 (Sta)*	TET, SXT	calf	O103
**STEC108**	1983	*Stx1*	Susceptible	calf	O139
**STEC109**	1986	*Stx1* (LT-II)	AMP, TET, STREP, SXT	calf	O1
**STEC110**	1988	*Stx1; Stx2*	AMP, TET, STREP, SXT	calf	O144
**STEC111**	1989	*Stx1*	Susceptible	calf	O123
**STEC112**	1989	*Stx2* (*Eae*)	Susceptible	calf	Rugous
**STEC113**	1989	*Stx1 (Sta)*	Susceptible	calf	O123
**STEC114**	1994	*Stx1*	TET	calf	O117
**STEC115**	1995	*Stx1*	TET	calf	O123
**STEC116**	1996	*Stx1* (LT-II)	AMP, TET, STREP, SXT	calf	O103
**STEC117**	1993	*Stx1*	AMP	calf	O10
**STEC118**	1996	*Stx1* (LT-II)	AMP	calf	O146
**STEC119**	1998	*Stx1* (LT-II)	AMP	calf	O146
**STEC120**	1999	*Stx1* (LT-II)	CF, QUIN, OXT	calf	O146
**STEC121**	1994	*Stx1 (Sta)*	AMP, TET, STREP, SXT	calf	O117
**STEC122**	1995	*Stx1*	AMP, STREP, OXT, CF, QUIN, GEN	calf	O23
**STEC123**	1996	*Stx1* (LT-II)	TET	calf	O146
**STEC124**	1993	*Stx1*	Susceptible	calf	O124
**STEC125**	1996	*Stx1 (Sta)*	AMP, TET, STREP, SXT	calf	O117
**STEC126**	2003	*Stx1 (Sta)*	TET, AMP	calf	O103
**STEC127**	2008	*Stx1*	AMP, TET, STREP, SXT	calf	O101
**STEC128**	2006	*Stx1* (LT-II)		calf	O146
**STEC129**	2002	*Stx1*	AMP, STREP, OXT, CF, QUIN, GEN	calf	O101
**STEC130**	2002	*Stx1*	TET	calf	O139
**STEC131**	2005	*Stx1* (LT-II)	TET, AMP, QUIN, OXT, GEN	calf	O124
**STEC132**	2008	*Stx1*	CF, QUIN, OXT	calf	O139
**STEC133**	2003	*Stx1*	AMP, STREP, OXT, CF, QUIN, GEN	calf	O146
**STEC134**	2008	*Stx1* (LT-II)	AMP, TET, CF, STREP, OXT, NA, GEN	calf	O124
**STEC135**	2006	*Stx1*	TET	calf	O101
**STEC136**	2002	*Stx1*	Susceptible	calf	O139
**STEC137**	1994	*Stx1*	Susceptible	calf	O139
**STEC138**	1995	*Stx1* (LT-II)	AMP, CF, STREP, OXT, SXT, GEN	calf	O124
**STEC139**	1996	*Stx1* (LT-II)	AMP	calf	O139

**STEC140**	1993	*Stx1* (LT-II)	Susceptible	calf	O23
**STEC141**	1996	*Stx2 (Sta)*	AMP, CF, TET, STREP, OXT, SXT, GEN	calf	O144
**STEC142**	1998	*Stx1*	Susceptible	calf	O101
**STEC143**	1999	*Stx2 (Sta)*	Susceptible	calf	O144
**STEC144**	1994	*Stx1*	Susceptible	calf	O117
**STEC145**	1995	*Stx1* (LT-II)	Susceptible	calf	O103
**STEC146**	1996	*Stx1* (LT-II)	Susceptible	calf	O144
**STEC147**	1993	*Stx1* (LT-II)	Susceptible	calf	Rugous
**STEC148**	1996	*Stx1*	Susceptible	calf	Rugous
**STEC149**	1988	*Stx2 (Sta)*	Susceptible	calf	O144
**STEC150**	1982	*Stx1*	Susceptible	calf	Rugous
**STEC151**	1983	*Stx1* (LT-II)	Susceptible	calf	ONT
**STEC152**	1988	*Stx1* (LT-II)	Susceptible	calf	O117
**STEC153**	1982	*Stx1*	Susceptible	calf	O139
**STEC154**	1983	*Stx1*	Susceptible	calf	O139
**STEC155**	1988	*Stx1* (LT-II)	Susceptible	calf	Rugous
**STEC156**	2000	*Stx1*	TET	calf	Rugous
**STEC157**	2008	*Stx1* (LT-II)	TET, AMP	calf	O124
**STEC158**	2001	*Stx1*	TET	calf	O139
**STEC159**	2003	*Stx1* (*Eae*)	TET	piglet	O26
**STEC160**	1994	*Stx1*	Susceptible	piglet	O101
**STEC161**	1995	*Stx1*	Susceptible	piglet	O101
**STEC162**	1996	*Stx1* (LT-II)	Susceptible	piglet	O124
**STEC163**	1993	*Stx1*	Susceptible	calf	O146
**STEC164**	1996	*Stx1*	Susceptible	calf	O153
**STEC165**	1998	*Stx1*	Susceptible	calf	O139
**STEC166**	1999	*Stx2* (*Eae*)	Susceptible	calf	Rugous
**STEC167**	1994	*Stx2* (*Eae*)	Susceptible	calf	Rugous
**STEC168**	1995	*Stx2* (*Eae*)	Susceptible	calf	Rugous
**STEC169**	1996	*Stx1*	Susceptible	calf	O124
**STEC170**	1993	*Stx1 (Sta)*	Susceptible	calf	O144
**STEC171**	1996	*Stx1* (*Eae*)	Susceptible	calf	O23
**STEC172**	2012	*Stx1*	TET	calf	O101
**STEC173**	2011	*Stx1* (LT-II)	AMP, CF, TET, STREP, OXT, SXT, GEN	calf	O124
**STEC174**	2015	*Stx1*	TET, AMP, CF	calf	O101
**STEC175**	2016	*Stx1* (*Eae*)	TET, STREP, GEN	calf	O23

References

1. Mazel, D., B. Dychinco, V.A. Webb, and J. Davies. 2000. Antibiotic resistance in the ECOR Collection: integrons and identification of a novel *aad* gene. Antimicrob. Agents Chemother. 44:1568–1574.
2. Ojeniyi, B., P. Ahrens, and A. Meyling. 1994. Detection of fimbrial and toxin genes in *Escherichia coli* and their prevalence in piglets with diarrhoea. The application of colony hybridization assay, polymerase chain reaction and phenotypic assays. J. Vet. Med. 41:49–59.
3. Pardon, B., B. Catry, J. Dewulf, D. Persoons, M. Hostens, K. De Bleecker, and P. Deprez. 2012. Prospective study on quantitative and qualitative antimicrobial and anti-inflammatory drug use in white veal calves. J. Antimicrob. Chemother. 67:1027–1038.
4. Picco, N.Y., F.E. Alustiza, R.V. Bellingeri, *et al.* 2015. Molecular screening of pathogenic *Escherichia coli* strains isolated from dairy neonatal calves in Cordoba province, Argentina. Rev. Argent. Microbiol. 47:95–102.
5. Radhouani, H., P. Poeta, G. Igrejas, A. Goncalves, L. Vinue, and C. Torres. 2009. Antimicrobial resistance and phylogenetic groups in isolates of *Escherichia coli* from seagulls at the Berlengas nature reserve. Vet. Rec. 165:138–142.
6. Randall, L.P., S.W. Cooles, M.K. Osborn, L.J. Piddock, and M.J. Woodward. 2004. Antibiotic resistance genes, integrons and multiple antibiotic resistances in thirty-five serotypes of *Salmonella enterica* isolated from humans and animals in the UK. J. Antimicrob. Chemoth. 53:208–216.
7. Roosendaal, B., W. Gaastra, and F.K. de Graaf. 1984. The nucleotide sequence of the gene encoding the K99 subunit of enterotoxigenic *Escherichia coli*. FEMS Microbiol. Lett. 22:253–258.
8. Saenz, Y., L. Brinas, E. Domınguez, J. Ruiz, M. Zarazaga, J. Vila, and C. Torres. 2004. Mechanisms of resistance in multiple-antibiotic resistant *Escherichia coli* strains of human,

animal, and food origins. Antimicrob. Agents Chemother. 48:3996–4001.

9. Shimizu, M., T. Sakano, J. Yamamoto, and K. Kitajima. 1987. Incidence and some characteristics of fimbriae FY and 31A of *Escherichia coli* isolates from calves with diarrhea in Japan. Microbiol. Immun. 31:417–426.

10. Toro, C.S., M. Farfan, I. Contreras, O. Flores, N. Navarro, G.C. Mora, and V. Prado. 2005. Genetic analysis of -resistance determinants in multidrug-resistant *Shigella* strains isolated from Chilean children. Epidem. Infect. 133:81–86.
